# Increased bronchiolitis burden and severity after the pandemic: a national multicentric study

**DOI:** 10.1186/s13052-024-01602-3

**Published:** 2024-02-13

**Authors:** Sergio Ghirardo, Nicola Ullmann, Alessandro Zago, Michele Ghezzi, Marta Minute, Barbara Madini, Enza D’Auria, Cecilia Basile, Francesca Castelletti, Federica Chironi, Agata Capodiferro, Beatrice Andrenacci, Francesco Maria Risso, Salvatore Aversa, Laura Dotta, Antonella Coretti, Anna Chiara Vittucci, Raffaele Badolato, Alessandro Amaddeo, Egidio Barbi, Renato Cutrera

**Affiliations:** 1 Department of Medicine, Surgery and Health Sciences, University of Trieste, via dell’Istria 65/1, Trieste, Italy; 2 Pediatric Pulmonology and Cystic Fibrosis Unit, Respiratory Intermediate Care Unit, Sleep and Long-Term Ventilation Unit, Bambino Gesù Children’s Hospital, IRCCS, Rome, Italy; 3 Department of Pediatrics, Buzzi Children’s Hospital, Milan, Italy; 4 Ospedale Regionale Ca Foncello Treviso, Treviso, Italy; 5 S.C. Pediatria Pneumoinfettivologia Fondazione IRCCS Cà Granda Ospedale Maggiore Policlinico, Milan, Italy; 6 Department of Clinical Sciences and Community Health, Università degli Studi di Milano, Milan, 20122 Italy; 7 Department of Clinical and Experimental Medicine, Section of Pediatrics, University of Pisa, Pisa, Italy; 8 Neonatal Intensive Care Unit, Children’s Hospital, ASST Spedali Civili, Brescia, Italy; 9 Department of Pediatrics and “A. Nocivelli” Institute for Molecular Medicine, Department of Clinical and Experimental Sciences, ASST Spedali Civili of Brescia, University of Brescia, Brescia, Italy; 10 Institute for Maternal and Child Health-IRCCS “Burlo Garofolo”, Trieste, 34137 Italy

**Keywords:** Bronchiolitis, Post-phandemic viral epidemiology, Bronchiolitis severity, Coinfections, COVID-19 and bronchiolitis

## Abstract

**Background:**

The coronavirus 2019 (COVID-19) related containment measures led to the disruption of all virus distribution. Bronchiolitis-related hospitalizations shrank during 2020–2021, rebounding to pre-pandemic numbers the following year. This study aims to describe the trend in bronchiolitis-related hospitalization this year, focusing on severity and viral epidemiology.

**Methods:**

We conducted a retrospective investigation collecting clinical records data from all infants hospitalized for bronchiolitis during winter (1st September-31th March) from September 2018 to March 2023 in six Italian hospitals. No trial registration was necessary according to authorization no.9/2014 of the Italian law.

**Results:**

Nine hundred fifty-three infants were hospitalized for bronchiolitis this last winter, 563 in 2021–2022, 34 in 2020–2021, 395 in 2019–2020 and 483 in 2018–2019. The mean length of stay was significantly longer this year compared to all previous years (mean 7.2 ± 6 days in 2022–2023), compared to 5.7 ± 4 in 2021–2022, 5.3 ± 4 in 2020–2021, 6.4 ± 5 in 2019–2020 and 5.5 ± 4 in 2018–2019 (*p* < 0.001), respectively. More patients required mechanical ventilation this winter 38 (4%), compared to 6 (1%) in 2021–2022, 0 in 2020–2021, 11 (2%) in 2019–2020 and 6 (1%) in 2018–2019 (*p* < 0.05), respectively. High-flow nasal cannula and non-invasive respiratory supports were statistically more common last winter (*p* = 0.001 or less). RSV prevalence and distribution did not differ this winter, but coinfections were more prevalent 307 (42%), 138 (31%) in 2021–2022, 1 (33%) in 2020–2021, 68 (23%) in 2019–2020, 61 (28%) in 2018–2019 (*p* = 0.001).

**Conclusions:**

This study shows a growth of nearly 70% in hospitalisations for bronchiolitis, and an increase in invasive respiratory support and coinfections, suggesting a more severe disease course this winter compared to the last five years.

## Background

In high-income countries, bronchiolitis represents the leading cause of infant admission at the hospital [1], causing a significant burden for emergency departments (ED) and paediatric wards due to clinical care requirements, number of cases and seasonal distribution with a typical winter peak led by respiratory syncytial virus (RSV) [2, 3]. Bronchiolitis may often induce the need for respiratory or nutritional support representing a leading cause of admission to paediatric or neonatal intensive care units (ICU) in the most severe cases [4]. During the SARS-COV-2 pandemic, social distancing and masking measures profoundly changed viruses’ distribution and seasonal patterns, determining a nearly complete disappearance of viral bronchiolitis with a very different seasonality [5–7]. The withdrawal of these measures resulted in an unusual seasonal distribution of hospitalization for bronchiolitis the following year with a precocious and higher peak but a similar overall number of cases due to a faster reduction [8, 9]. Surprisingly, after the pandemic there was also an increase in the use of high flow nasal cannula (HFNC) and in the admission to ICU [8]; whether this change is attributable to a more severe disease or a change in the attitude of physicians after the pandemic is to be determined.

The study aims to establish the trend in need for bronchiolitis-related hospitalization, severity and microbiological characteristics during the last five years in Italy.

## Methods

We conducted a retrospective study in six Italian hospitals: Institute for Maternal and Child Health—IRCCS “Burlo Garofolo”, Trieste; Bambino Gesù Children’s Hospital IRCCS, Rome; Vittore Buzzi Children’s Hospital, University of Milan, Milan; ASST Spedali Civili, Brescia; Ca’ Granda Ospedale Maggiore Policlinico, Milan; Ospedale Regionale Ca Foncello, Treviso. We retrospectively reviewed medical records of all children (< 1 year) hospitalized between 1st September and 31st March (winter season) of every subsequent year since September 2018. We included for the analysis all the patients with a final diagnosis of bronchiolitis at discharge as reported by the clinical records ICD 9 codes 466.11 and 466.19.

For every patient, we collected the following data: age, sex, gestational age and weight at birth and underlying comorbidities, defined as any disease already present at the bronchiolitis onset. The comorbidities were grouped, according with the literature [10], into the following five groups: ex-preterm, patients with neuromuscular disease, patients with congenital heart disease, patients with chronic lung illness, patients with immunodeficiency. We collected data on virologic results of polymerase chain reaction (PCR) panels capable of detecting at least 5 viruses nasal or pharyngeal swabs or aspirates, date of hospitalisation and discharge, admission to the ICU and length of ICU stay, need for and days spent on oxygen therapy, high flow nasal cannula (HFNC), non-invasive and invasive respiratory support, respectively.

### Statistical analysis

We reported discrete variables as numbers and percentages. We evaluated continuous variables for normality graphically and using the Shapiro–Wilk test. Normally distributed continuous variables were registered as mean and standard deviation, whereas we reported non-normally distributed continuous variables as median with the first and third quartile. We compared the various winter seasons 2018–2019, 2019–2020, 2020–2021, 2021–2022 and 2022–2023 using the One-Way ANOVA with post hoc comparisons using Tukey’s HSD at alpha = 0.05. For non-parametric group analysis, Kruskal–Wallis ANOVA with post hoc multiple group Bonferroni correction with an alpha of 0.05. We compared the last winter season (2022–2023) with the previous one using the Chi-square test for discrete data and the Student T-test for continuous variables. We employed the log-rank test for the length analysis, such as lengths of stays and the number of days needing every kind of respiratory support. We considered statistically significant *p* values < 0.05.

### Outcomes

The primary outcome was the number of hospitalizations for bronchiolitis from the 1st of September to the 31st of March 2022–2023 (period defined as winter season) compared to the previous four years. Secondary results were the length of in-hospital and ICU stays, the need for oxygen; HFNC; non-invasive respiratory support (NIRS); or mechanically ventilation patients (MV). NIRS was defined as the use of either continuous positive airway pressure (CPAP) and/or non-invasive ventilation (NIV). We collected data about the number of days of every respiratory support. We registered the virological results and the type of test performed, in particular, referring to RSV and coinfections. We reported the monthly distribution of hospitalization in every five seasons.

## Results

### Hospitalization rate and patients’ characteristics

Between the 1st of September 2022 and the 31st of March 2023 (winter season), a total of 953 infants were hospitalised for bronchiolitis at the six hospitals, 563 in the same period in 2021–2022, 34 in 2020–2021, 395 in 2019–2020 and 483 in 2018–2019. As shown in Table 1, gestational age and weight at birth did not differ significantly from a statistical perspective. However, As shown in Table 1, gestational age and weight at birth did not significantly differ, but the age at admission was remarkably higher during the 2020–2021 winter season (*p* < 0.001). Comorbidities did not differ between the various winter seasons. All population data are reported in Table 1.

**Table 1:** Patients’ characteristics at the admission

	Age at admission, days (mean ± SD)	Weight at birth, grams (mean ± SD)	Gestational age at birth, weeks (mean ± SD)	Comorbidities (n, %) all comorbidities; preterm, congenital heart disease, chronic lung illness, neuromuscular disease patients/immunodeficiencies
2018–2019 *N* = 483	89 ± 74	3163 ± 649	38 ± 2.3	83 (17%); 52/22/8/1/0
2019–2020 *N* = 395	86 ± 75	3121 ± 613	38 ± 2.4	70 (18%); 43/13/13/0/1
2020–2021 *N* = 34	142 ± 114	2933 ± 902	38 ± 3.3	10 (29%); 4/2/4/0/0
2021–2022 *N* = 563	79 ± 74	3185 ± 630	38 ± 2.6	154 (27%); 51/35/14/4/1
2022–2023 *N* = 953	89 ± 78	3155 ± 617	38 ± 2.3	168 (18%); 109/43/13/3/0
*p*-value	*p* < 0.001	*p* = 0.30	*p* = 0.38	*p* < 0.001

*SD* Standard deviation

### Length of hospitalization and ICU admissions

During the 2022–2023 winter season patients, spent in hospital an average of 7.2 ± 6 days hospitalised, 5.7 ± 4 days in the 2021–2022 winter season, 5.3 ± 4 days in the 2020–2021 winter season, 6.4 ± 5 days in the 2019–2020 winter season and 5.5 ± 4 days in the 2018–2019 winter season, respectively. The length of stay was significantly longer on the 2022–2023 and 2019–2020 winters than the others (*p* < 0.001).

In the 2022–2023 winter, 210 patients were admitted to ICU for cumulative 1349 days, 122 patients (696 days) in the 2021–2022 winter, 6 patients (17 days) in 2020–2021, 84 patients (437 days) in 2019–2020 and 92 patients (435 days) in the 2018–2019 winter. The percentage of subjects admitted and the length of stay at ICU was not statistically different.

### Need for respiratory support

Chest X-ray was performed in 401 (42%) patients in 2022–2023, 345 (61%) in 2021–2022, 15 (44%) in 2020–2021, 200 (51%) in 2019–2020 and 227 (47%) in 2018–2019. HFNC, NIRS and MV were statistically more common in the last winter than the previous ones (*p* = 0.001 or less); the prevalence of oxygen use was statistically significantly higher during the 2021–2022 winter season. Data about respiratory supports and ICU needs are reported in Table 2.

**Table 2:** Prevalence and length of respiratory support and intensive care unit admission

	Oxygen		HFNC		NIRS		MV		ICU	
	Patients n (%)	Days mean ± SD	Patients n (%)	Days mean ± SD	Patients n (%)	Days mean ± SD	Patients n (%)	Days mean ± SD	Number (percentage)	Days mean ± SD
2018–2019	253 (52%)	4.1 ± 3	141 (29%)	4.1 ± 2	68 (14%)	3.6 ± 2	6 (1%)	5.2 ± 2	86 (18%)	5 ± 3.5
2019–2020	227 (57%)	4.3 ± 3	115 (29%)	4.5 ± 2	69 (17%)	3.4 ± 2	11 (2%)	5.3 ± 2	84 (21%)	5.2 ± 3.2
2020–2021	16 (47%)	3.6 ± 3	10 (29%)	3.9 ± 5	5 (15%)	2.0 ± 1	0	NA	6 (18%)	2.8 ± 1.6
2021–2022	414 (73%)	4.6 ± 3	300 (53%)	4.5 ± 2	99 (18%)	4.0 ± 3	6 (1%)	4.0 ± 3	122 (22%)	5.7 ± 3.4
2022–2023	505 (53%)	4.4 ± 3	585 (61%)	4.4 ± 3	217 (23%)	3.7 ± 2	38 (4%)	6.3 ± 5	210 (22%)	6.4 ± 6.5
*P*-value	*p* < 0.001	*p* = 0.18	*p* < 0.001	*p* = 0.59	*p* = 0.001	*p* = 0.29	*p* < 0.001	*p* = 0.71	*p* = 0.4	*p* = 0.068

*HFNC* High-Flow Nasal cannula, *NIRS* Non-Invasive Respiratory Supports, *ICU* Intensive Care Unit, *MV* Mechanically ventilated

### Virological findings

**Fig. 1:**
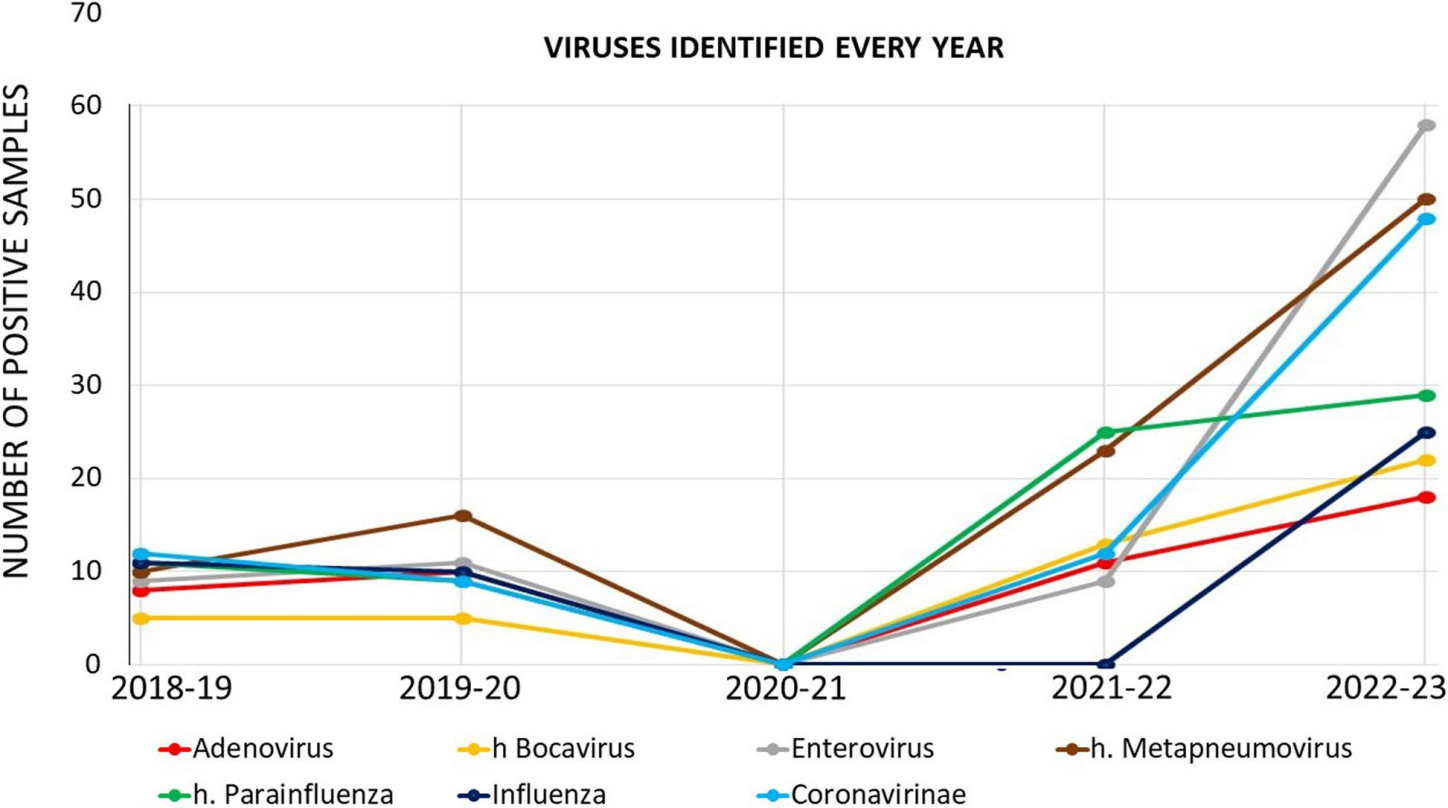
number of the seven mostly frequently identified viruses in the five considered winter season (1st September 31st March). Rhinovirus data are not reported here to improve the readability of the graph. The category Coronavirinae contains the four human coronaviruses together with the SARS-CoV-2

In the 2018–2019 winter season, 323/476 (68%) patients had a positive nasopharyngeal swab test for RSV, 286/393 patients (72%) in 2019–2020, 3/34 patients (8%) in 2020–2021, 457/560 patients (81%) in 2021–2022 and 749/946 patients (79%) in 2022–2023; the prevalence of RSV was statistically notably lower during 2020–2021 winter season (*p* < 0.001).RSV was found together with another virus or more than one viruses (coinfection and RSV positivity coinfection & RSV +)more frequently during the latter two winter seasons *p* = 0.001. rhinovirus was the second most frequently identified virus on polymerase chain reaction (PCR) panels in each season, with a statistically substantial higher prevalence during 2020–2021 and the next two winter seasons (*p* < 0.001). Coinfections between viruses in patients RSV negative occurred more frequently during the last two winter seasons (*p* < 0.001). Influenza virus (A and B) was found in 25 patients in 2022–2023, 0 patients in 2021–2022, 0 patients in 2020–2021, 10 patients in 2019–2020, 11 patients in 2018–2019; this reduction in 2020–2021 and 2021–2022 is statistically significant *p* = 0.002. Parainfluenza viruses were identified in 29 patients in 2022–2023, 25 patients in 2021–2022, 0 patients in 2020–2021, 9 patients in 2019–2020, 11 patients in 2018–2019; *p* = 0.15. Apart from RSV and rhinovirus We graphically reported information about the trends of the seven mostly frequently identified viruses in Fig. 1; rhinovirus data are reported in Table 2 to improve the readability of the graph. In Fig. 1 the category Coronavirinae contains the four human coronaviruses together with the SARS-CoV-2 virus that was identified in 3 cases in 2021–2022 and 2022–2023 respectively.

Among the 38 patients who required MV during the 2022–2023 winter, 15 (39%) presented coinfections; in 13 of such cases, RSV was isolated, 3 out of the 11 MV presented coinfections in 2019–2020 in 2 of these cases RSV was involved; none of the MV patients during 2021–2022 and 2018–2019 winter presented coinfections. Influenza virus was found in 9 cases (2.4% of all tested) in the 2018–2019 winter season, 9 in 2019–2020 (2.6%), 0 in 2020–2021, 0 in 2021–2022 and 39 (5.4%) in 2022–2023; therefore, influenza was more commonly detected the last winter season (*p* = 0.009). Virological results are shown in Table 3.

**Table 3:** Virological results

	RSV + n/n tested (%)	RSV + among ICU patients (% of ICU patients)	n (%) tested with panels; Rhinovirus + n (% of tested)	Patients presenting coinfections & RSV + (% out of RSV +)	Coinfections in patients without RSV	Coinfected patients in ICU (% of ICU patients); RSV + among these patients n (%)	Coinfected among MV n (% out of MV); RSV + n(%)
2018–2019	323/476 (68%);	77/86 (89%)	371 (77%); 60 (16%)	47 (14%)	14 (4%)	19 (22%); 15 (17%)	0/6
2019–2020	286/393 (73%)	67/84 (80%)	347 (88%); 52 (15%)	48 (17%)	20 (6%)	18 (21%); 14 (17%)	3/11 (27%); 2 (18%)
2020–2021	3/34 (9%)	1/6 (15%)	29 (85%); 19 (65%)	1 (33%)	0	0; 0	0
2021–2022	457/560 (82%)	112/122 (92%)	387 (69%); 93 (22%)	97 (21%)	41 (10%)	21 (17%); 17 (14%)	0/6
2022–2023	749/946 (79%)	180/210 (86%)	715 (75%); 198 (27%)	185 (25%)	122 (17%)	68 (32%); 52 (25%)	15/38 (39%); 13 (34%)
*P*-value	*p* < 0.001	*p* < 0.001	*p* < 0.001; *p* < 0.001	*p* = 0.001	*p* < 0.001	*p* = 0.01; *p* = 0.08	

*PCR* Polymerase Chain Reaction, *RSV* + Respiratory Syncytial Virus positive, *ICU* Intensive Care Unit

**Fig. 2:**
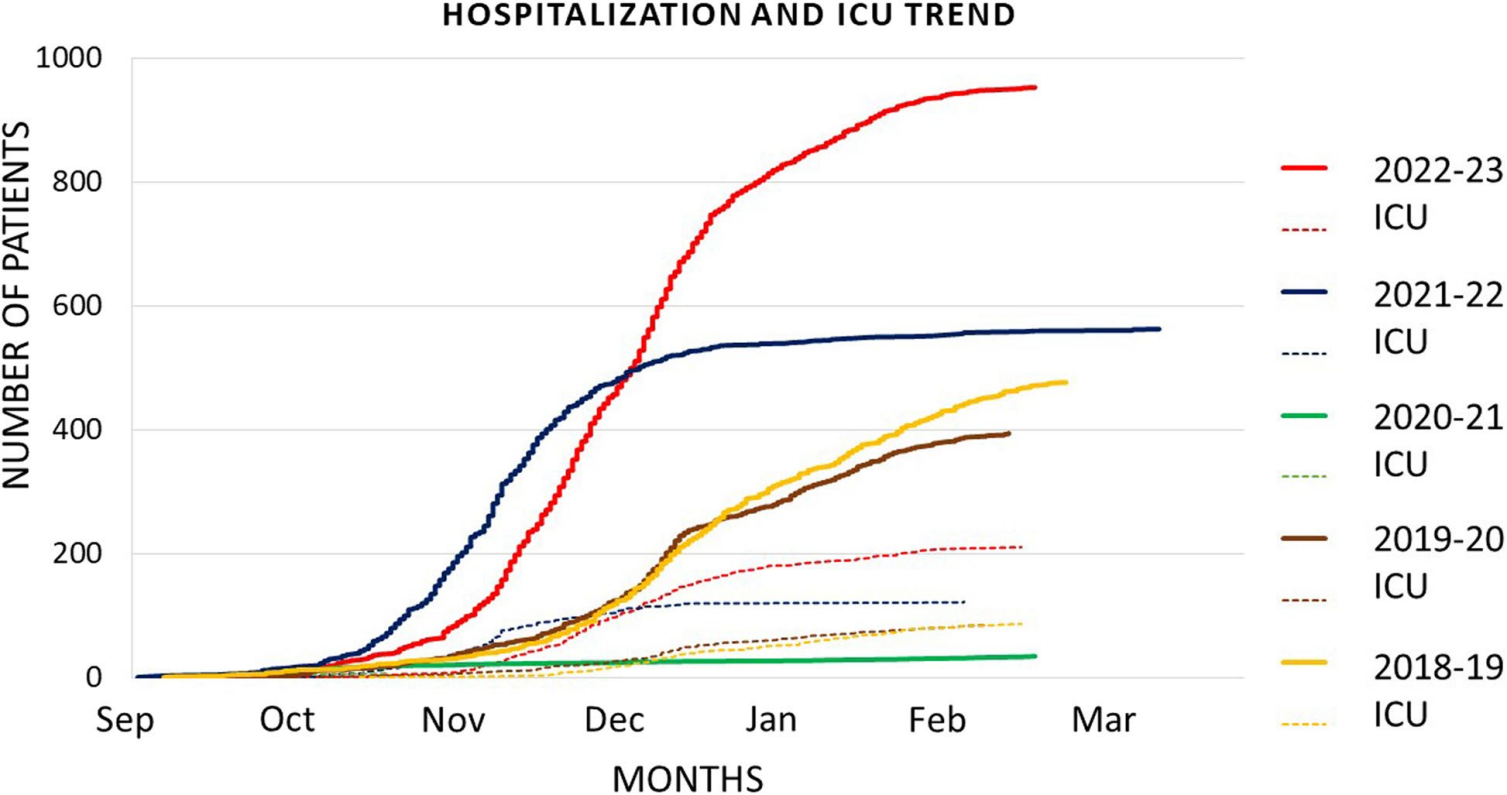
monthly distribution of hospitalizations and admission to the intensive care unit every winter season (1st September 31st March)

Applying the Log-rank test, we noticed statistically considerable anticipation in the hospitalizations for bronchiolitis in the 2021–2022 winter season (*p* < 0.001). Hospitalizations occurred after a mean of 110 ± 31 days since the 1st of September in the 2022–2023 season, 85 ± 30 days in the 2021–2022, 83 ± 60 days in the 2020–2021, 122 ± 35 days in the 2019–2020, 131 ± 39 in the 2018–2019 seasons, respectively. The hospitalisation trend in the various winter seasons is shown in Fig. 2.

## Discussion

This study describes a marked increase in the absolute number of hospitalizations for viral bronchiolitis during the last winter season, and the severity of disease course is defined as a higher percentage of ICU admissions and MV rates. In particular, the number of children who were hospitalized for viral bronchiolitis in the 2022–2023 winter was roughly double compared to the pre-pandemic seasons and presented a 70% increase compared to 2021–2022 winter season. Our study is the first one reporting such a post-pandemic abrupt increase in bronchiolitis-related hospitalisation and growing severity.

It could be due to the combined effect of the complete removal of restrictions on the 25th of March 2022 in Italy (dl n.24 22G00034) and to [11] the relaxation of self-imposed anti-infective rules by parents.

The abrupt and precocious fall in the number of patients presenting an RSV infection in the January of 2022 resulted in a more extended RSV-free period this year, leading to an enlarged gap between the possible last maternal RSV infection and the delivery. This phenomenon may have reduced the effectiveness of the transplacental immunoglobulin newborns’ coverage [12], causing a less effective passive immunisation of the infants [13], thus justifying a more severe disease presentation with an increased need for hospitalisation.

However, despite the different total amount of hospitalisation last winter, the monthly distribution of cases was much closer to the pre-pandemic period, thus suggesting a less naive population than what was speculated for the 2021–2022 winter and, therefore a slower spreading of RSV [14].

From this perspective, this increase may also be partially explained by a rise in coinfections. After the pandemic, the higher prevalence of coinfections of RSV and other viruses, such as rhinovirus, may result from the transmission of multiple viruses at once by the same subject that presents an immune debt [15]. The coinfection phenomenon in these patients is consistent with the co-dissemination of social contagion theory, which relies on the synergy between viruses rather than the competition if the patient encounters them quickly before an effective immunological response [16]. Influenza virus was completely absent through the 2020–2021 winter, probably due to social distancing and COVID-19 containment measures as already reported. On the contrary the absence of influenza virus in the 2021–2022 winter season in patients with bronchiolitis is in contrast with the partial resurgence of this particular virus reported in adults that year [17]. The last winter season influenza more than doubled compared to the baseline represented by the two pre-pandemic winter seasons. With this sort of delay in its resurgence influenza virus constitute a unicum in our study that is yet to be understood.

Another remarkable finding is the trend of influenza virus that was never detected in our patients during the containment measures and the following year [18] but spread during this winter season to reach a higher incidence compared to the pre-pandemic years.

Concerning disease severity, we noted that the percentage of subjects admitted to ICU, length of stay and the need for respiratory support presented a marked increase throughout the years. Firstly, last winter, the number of patients requiring intubation quadrupled compared to the previous years. HFNCs were utilized more frequently than oxygen (61% vs 53%). Remarkably, this winter, 80 patients received HFNC with room air in this series, possibly as a consequence of the endorsement given by the guideline to use HFNC to administer a minimal positive end-expiratory pressure [19], notwithstanding the weak evidence sustaining the efficacy of this device in preventing respiratory failure in bronchiolitis [20]. This unprecedented finding outside of clinical studies on HFNC raised concerns about the need for a more precise indication for the use of HFNC [21].

The trend toward a more aggressive approach to treating bronchiolitis has been debated recently. possibly motivated by the increased availability of relatively more advanced respiratory support outside the intensive care units [22–24]. In particular, the broad availability of high-flow nasal cannula seems to play a crucial role in this process, and it has been reported as paradoxically even leading to an increase in mortality [25–28]. A remarkable finding of our study is that despite the increased HFNC use this winter, the number of MV patients contextually reached its peak and the length of hospital stay was higher. Increased severity of the disease may determine these data. However, it may also support the hypothesis of a lack of efficacy of HFNC in pre-empting the need for MV, as recently reported, in contrast to previous findings [29, 30]. Remarkably, The HFNC use remained stable at 29% until the pandemic winter season and grew the following winter to 53% and 61% the past winter. This hit suggests that physicians may have developed a different attitude in treating respiratory distress more aggressively during the pandemic [31].

Although statistically significant, the raised NIRS use in our study is less pronounced than the massive augmented use of HFNC since the pandemic. On the opposite side to HFNC, the efficacy of NIRS to reduce the necessity for intubation is established [32]. Despite the extensive usage of these effective devices, we also registered an abrupt increase in the intubation rate this winter. The trend favouring more aggressive management of patients with bronchiolitis [33] cannot be addressed for the whole phenomenon, including intubations. It seems unlikely that an attitude change could be addressed for such an increase in NIRS usage and this significant growth in the intubation rate; moreover, although only close to the statistically significant figure, the length of stay in the ICU also extended this year. From our perspective, the data suggest that this year patients experienced, on average, a more severe disease, with a higher need for advanced respiratory support and intubation rate. The lack of a clear explanation of this newly discovered phenomenon might partly be a consequence of the expected lowering of maternal antibodies to the anticipation of the RSV season the past winter [13]. However, it should not be addressed for the whole phenomenon, and we speculated about the role of a likely immunological debt [15] due to the profound reduction of virus circulation during the pandemic [17]. On the other hand, the increase in hospitalization may be the consequence of an overall more severe course of the disease due to the “immunological debt” and the higher rate of coinfections in the 2022–2023 winter season that seems to play a role in disease severity in contrast with the pre-pandemic literature that reports similar bronchiolitis severity for patients with and without coinfections [26]. However, it could be speculated that patients from the previous literature were not coming from a background of lack of exposure to infections as the post-pandemic ones.

### Study strengths

It is a sizable multicentric study reporting data from more than 2400 patients with a relatively high viral identification coverage and the first study reporting on this particular winter season.

### Study limitations

The virological tests were not performed systematically due to the retrospective study’s retrospective nature. The viral PCR panels differ between various centres and changed at the same centre in these years. The study was performed only in Italian hospitals and only during the winter seasons (1st September to 31 of March) which may be a partial information due to the partial shift in RSV seasonality that we are experiencing as a result of the pandemic. We can only partially rule out possible misclassifications of patients in clinical records in attributing ICD9 codes.

The number of hospitalised cases and the bronchiolitis related burden this winter was well beyond the pre-pandemic period, raising concerns about the risks of exceeding vital support availability if we experience a further similar increase the following year. Therefore, we strongly suggest supporting handwashing and other containment measures for the whole familial nucleus of the infants.

## Conclusions

This study shows a significant increase in hospitalisations and disease severity for bronchiolitis in infants and invasive respiratory support in the 2022–2023 winter season. Coinfections have played a major role in the hypothesis of a post-pandemic immunological debt, but more data are needed to confirm our results.

## Data Availability

The datasets used and/or analysed during the current study are available from the corresponding author on reasonable request.

## Abbreviations

SARS-CoV-2: Severe acute respiratory syndrome-corona virus-2
COVID-19: Corona virus disease 19
ED: Emergency department
RSV: Respiratory syncytial virus
ICU: Intensive care unit
HFNC: High flow nasal cannula
PRC: Polymerase chain reaction
MV: Mechanically ventilated
SD: Standard deviation
NIRS: Non-invasive respiratory support
CPAP: Continuous positive airway pressure
NIV: Non-invasive ventilation
